# 

**Published:** 1923-02

**Authors:** 


### GOVERNMENT PUBLICATIONS.

Copies can be purchased through any bookseller or direct from H.M. Stationery Office, at Imperial House, Kingsway, W.C.2 ; 28 Abingdon Street, S.W.1 ; 37 Peter Street, Manchester ; 23 Forth Street, Edinburgh ; and 1 St. Andrew's Crescent, Cardiff. The prices quoted include postage.

### STATIONERY OFFICE PUBLICATIONS.

Scottish Board of Health.—Milk and Dairies, Scotland. The Milk (Special Designations) Order (Scotland), December 11, 1922, made by the Scottish Board of Health under Sections 3 and 14 of the Milk and Dairies (Amendment) Act, 1922. 2½d.

Ministry of Health.—National Health Insurance (Medical Benefit) Amendment Regulations (No. 2), 1922, December 11, 1922, made by the Minister of Health under the National Health Insurance Acts, 1911 to 1922. 2½d.

Ministry of Labour.—Disabled Ex-Service Men. Training and Employment. Select Committee's Report. (H.C. 170). 3½d.

Milk and Dairies, England. The Milk (Special Designations) Amendment Order, December 18, 1922, under Sections 3 and 6 of the Milk and Dairies Amendment Act, 1922. 1½d.

Ministry of Health.—Fees of Doctors called in by Midwives ; to Local Supervising Authorities under the Midwives' Acts ; December 20, 1922 (England). Pasteurised Milk ; to Sanitary Authorities (England and Wales) ; December 19, 1922.

### MINISTRY OF HEALTH.

Model By-laws : (b) Draft. Drainage of Existing Buildings. Dairies, Cowsheds, and Milk-shops. Model Regulations.

### STATUTORY RULES AND ORDERS.

(1346.) Nurses, England. Notice, December 5, 1922, given by the Minister of Health under Sec. 8 of the Nurses' Registration Act, 1919, of the Compilation of a Register.

Parliamentary Elections. Northern Ireland. The Government of Ireland (Election Laws Adaptation) (Northern Ireland) Order, December 6, 1922, adapting the Election Laws as to both elections to the Commons House of Parliament of the United Kingdom for constituencies in Northern Ireland, and Elections to the House of Commons of Northern Ireland.

(1377.) National Health Insurance (Insurance Committees) Amendment Regulations, December 22, 1922, made by the Minister of Health under Sec. 3 (2) of the National Health Insurance Act, 1921.

Allotments, Scotland. Allotments (Compulsory Leasing) Regulations, December 20, 1922.

Milk and Dairies, Scotland. Milk (Special Designations Amendment Order (Scotland), December 20, 1922, made by the Scottish Board of Health under Secs. 3 and 14 of the Milk and Dairies (Amendment) Act, 1922.

2. National Health Insurance (Medical Benefit) Consolidated Regulations, January 1, 1923, made by the Minister of Health under the National Health Insurance Acts, 1911 to 1922, 1s. 5½d., post free.

### PUBLIC HEALTH IN PARLIAMENT.

The following answers were given in the House of Commons, during the later part of the last Session, by Sir M. Barlow and Major Boyd Carpenter, speaking for the Minister of Health.

### HOUSING.

Borrowing Powers.—To Major Kelley : Local authorities are fully empowered under the Housing Acts to borrow money for the carrying out of housing schemes subject to the sanction of the Minister of Health, and such sanction is readily granted for satisfactory schemes. There is, generally speaking, no difficulty at the present time in raising capital for this purpose. The Public Works Loan Commissioners will consider applications from local authorities whose rateable value does not exceed £200,000, the rate of interest on loans made by the Commissioners for housing at present being 5 per cent. The large local authorities can generally make their own arrangements for borrowing on at least as favourable terms, and in addition they may borrow through the Public Works Loan Commissioners one-half the money raised in their areas by the sale of National Savings Certificates.

Local Activity.—To Mr. Albert Bennett : According to the information in the possession of my Right Hon. Friend, local authorities generally have shown great activity in dealing with the housing problem in its various aspects. They have during the last few years concentrated on the provision of new houses. A considerable programme of slum clearance is now being undertaken by various authorities with financial assistance from the Exchequer.

Sites (Cost).—To Mr. Mardy Jones : The total area of land acquired in connection with housing schemes is approximately 48,800 acres. The total cost is £9,272,000, being an average of £190 per acre.

### NATIONAL HEALTH INSURANCE.

Medical Service.—To Mr. Rhys Davies : Insurance Committees are required to investigate all specific complaints of failure on the part of insurance practitioners to carry out their obligations. Reports of all these investigations are forwarded to the Ministry of Health, and, where necessary, disciplinary action is taken. The question how far the service could be improved without unduly increasing the cost is under consideration in connection with the revision of the present agreement as to the capitation fee, which expires at the end of next year.

Medical Referees.—To Mr. Rhys Davies : The number of whole-time medical officers who, in addition to their own duties, are employed as medical referees is thirty-five. The number of insured persons referred to them during the financial year 1921-22, when the sphere of their operations was limited, was 81,762, of whom 47,404 attended for examination. Since the referee service was extended, during the present financial year, to cover the whole country, the number of references has increased to such an extent as to necessitate the employment of a certain number of part-time referees in addition.

Panel Doctors.—To Mr. Gould : The limit of the number of insured persons who an insurance practitioner may have on his list is fixed for each area jointly by the Insurance Committee and the Medical Panel Committee. No practitioner working single-handed may have more than 3,000 persons on his list, and in a considerable number of areas a lower maximum of 2,500 or 2,000 has been fixed. Taking the country as a whole, the average number of insured persons per doctor is approximately 1,000, and the corresponding remuneration, including mileage payments, would be slightly less than £500 a year. Insurance practitioners are expected to give the same standard of treatment, within the scope of their terms of service, to insured persons as they would to private patients.

Approved Societies (Surplus).—To Viscount Ednam : The total disposable surplus of Approved Societies and Branches in England disclosed as the result of the First Valuation was about £8,000,000, the major portion of which the members elected to take in the form of an increase of the ordinary cash benefit. Of the balance a sum of £950,596 has been allocated by certain societies for payments to hospitals and convalescent homes during the period from July, 1921, to July, 1926.

Treatment by Private Doctors.—To Mr. Gould : An insured person who desires to be treated by a practitioner not under agreement with an Insurance Committee may apply to the Insurance Committee for the area in which he resides for permission to make his own arrangements. If permission is granted, a contribution will be made by the Committee towards the cost of the treatment so obtained. The Insurance Committee has full discretion in considering applications of this nature. There is no provision in the Acts for any reduction in the contribution payable by or in respect of an insured person on the ground that he does not take advantage of the arrangement made under these Acts to provide him with medical treatment.

### INFECTIOUS DISEASES.

Mortality.—To Mr. Cecil Wilson : Notifications and deaths in the decennium 1911-1920.

| | London. | | | Remainder of England and Wales. | | |
|---|---|---|---|---|---|---|
| | Cases notified. | Deaths. | Deaths per 1,000 cases. | Cases notified. | Deaths. | Deaths per 1,000 cases. |
| Scarlet fever .. | 138,744 | 1,878 | 14 | 870,169 | 14,959 | 17 |
| Enteric fever .. | 5,851 | 1,008 | 172 | 60,486 | 11,419 | 189 |
| Small-pox | 170 | 23 | 135 | 1,293 | 111 | 86 |

Figures for port sanitary districts are included in the above table, but those for non-civilian are excluded from 1915 onwards, as they could be given only for England and Wales as a whole.

Vaccination.—To Dr. Watts : My Right Hon. Friend is keeping continuously before him the question of the necessity, or otherwise, of amending the Vaccination Acts, but he is not prepared at this stage of the present outbreak to introduce legislation.

### MISCELLANEOUS.

Milk (Pasteurisation).—To Sir John Leigh: My Right Hon. Friend is aware that there is some distrust of Pasteurisation as now practised, partly due to the high temperature processes frequently adopted commercially. There is no evidence that Pasteurisation properly conducted is capable of destroying the essential quatities or constituents of milk. The Minister of Health trusts that the steps being taken by his Department in regard to the milk supply will conduce to the provision of a wholesome milk at reasonable prices.

Ex-Service Men.—To Mr. Robert Richardson : No obstacles are placed by the Ministry of Pensions or the Ministry of Health in the way of the discharge of ex-service men and other inmates of asylums. This is not a matter in which either Department intervenes. Ex-service patients who have been accepted by the Ministry of Pensions and classified as service patients are upon the footing of private patients, and can be discharged under the provisions of Section 72 of the Lunacy Act, unless a certificate is given by the medical officer of the institution to the effect that the patient is dangerous and unfit to be at large. Other patients can only be discharged by the Visiting Committee, in whose discretion the matter rests.

Lunacy and Mental Deficiency.—To Mr. Frederick Roberts : Under Section 271 of the Lunacy Act, 1890, Visiting Committees are empowered to receive private patients into Asylums upon such terms as they may think fit. The Hon. Member is under a misapprehension in suggesting that their jurisdiction is limited to pauper patients.

Use of Insulin.—To Sir William Bull : So far from creating obstacles, the Medical Research Council are doing everything in their power to accelerate the production and use of Insulin in the treatment of diabetes. My Right Hon. Friend's assertion that the remedy has passed the experimental stage is not in accord with the experience of other countries or the best scientific opinion here. There is no question of any Government monopoly, and the suggestion which he makes as to manufacture [that licences should be granted to authorised persons] is on the lines which the Medical Research Council have been in fact diligently pursuing for some time past.

### RECENT APPOINTMENTS.

### Medical Officers, Chaplains, etc.

Barr, The Rev. H. L. N., Chaplain of the Swinton Moral and Industrial School.

Corkey, I. W., M.A., M.D., M.Ch., F.R.C.S., surgeon specialist for the London area, Ministry of Pensions; hon. assistant surgeon, East Sussex Hospital.

Craig, Georgina Scott; Assistant Medical Officer, Cheshire Education Committee.

Fairley, J., M.D., D.P.H., Assistant Medical Officer of Health, Portsmouth Co. Borough; County Medical Officer, Isle of Wight County Council (Glasgow University).

Gamlin, Raymond, M.A.. M.B., B.S. (Camb.), M.R.C.S., L.R.C.P. (Lond.), D.P.H., M.Hy. (Liverpool), Assistant M.O.H. for the Port and City of Liverpool; Assistant Medical Officer of Health (Tuberculosis and Venereal Diseases Clinical Officer), County Borough of Wallasey.

Gross, Malcolm, M.B., B.S., D.Ph., Assistant Medical Officer of Health for West Suffolk.

Hildyard, Gerald M. T., K.C., Master in Lunacy.

Jackson, Major Raphael; Secretary, Queen Mary's Hospital for the East End.

Jaffe, Louis; Senior Resident Medical Officer of the Jewish Hospital, London.

Lloyd, Major-General O. E. P., V.C.; Colonel-Commandant, Royal Army Medical Corps.

Mackintosh, Jean M., M.B., Ch.B., D.P.H. (R.C.P.S. Edin.), House Surgeon, Scarborough Hospital and Dispensary, Assistant Medical Officer of Health and Assistant Schools Medical Officer, Borough of Llanelly. (Glasgow University.)

Shea, Bernard Frederick, M.B., B.Ch., B.A.O., House Surgeon, Queensbury and District General Infirmary (National University in Dublin).

Stones, G. Fowler, Medical Officer, Royal Watson Hospital; Medical Officer, Epsom.

Thynne, Mildred A., Assistant Medical Officer for Maternity and Child Welfare Work, Bermondsey Borough Council.

### Public Health and Hospital Appointments.

(The brackets following the appointment indicate the institution at which the official was trained.)

Bailey, M. E., C.M.B., Matron, Innes Hopkins Memorial Home, Ryton-on-Tyne; Matron, Orthopædic Hospital, Stoke-on-Trent (North Staffordshire Infirmary and Eye Hospital, Stoke-on-Trent).

Beaghen, Annie C., C.M.B., Health Visitor and School Nurse, Borough of Accrington (Victoria Hospital, Blackburn).

Butler, Elsie W. (Queen Victoria Jubilee Nurses' Institute); Assistant Superintendent at Brighton.

Cohen, Kathleen Marion, Emergency Health Visitor and School Nurse for Staffordshire; Health Visitor, Lincoln Nursing Association (Holborn and Finsbury Hospital, Archway Road, N.).

Cooper, Norah Marion, Assistant Superintendent and Health Visitor, M. and C.W., Finsbury Borough Council; Health Visitor and School Nurse, County Council of Middlesex.

Darbyshire, R. E., R.R.C., Lady of Grace of the Order of St. John of Jerusalem, Kaisar-i-Hind Medallist, Chief Lady Superintendent, Lady Minto's Indian Nursing Association; Matron of University College Hospital. (St. Thomas's Hospital.)

Dowdy, Miss (Queen Victoria Jubilee Nurses' Institute, Scotland), Second Assistant, Glasgow.

Fudge, Gladys H., Night Sister and Assistant Matron, City Mental Hospital, Nottingham; Health Visitor, City of Leicester (North Evington Infirmary, Leicester).

Halliway, Dorothy, Infectious Diseases and V.D. Nurse, Lincolnshire Nursing Association (Royal Infirmary, Hull, and Monsall Fever Hospital).

Johnson, Beatrice M. (Queen Victoria Jubilee Nurses' Institute), Superintendent at Reading.

Jones, Florence S., House Sister, Royal Victoria Infirmary, Newcastle-on-Tyne: Matron, Memorial Cottage Hospital, Wickham, Co. Durham (Royal Victoria Infirmary, Newcastle-on-Tyne).

Laidlaw, Agnes, Theatre Sister, Birmingham and Midland Ear and Throat Hospital; Matron, Birmingham and Midland Ear and Throat Hospital.

MacEwing, C. M. (Queen Victoria Jubilee Nurses' Institute, Scotland), Assistant Superintendent and Health Visitor, Ayrshire.

Maclachlan, R. (Queen Victoria Jubilee Nurses' Institute, Scotland), Assistant Health Visitor and Emergency Nurse, Morayshire.

Newton, Marion, Sister Tutor, St. Chad's Hospital, Birmingham; Matron, Tamworth Hospital (Burton-on-Trent).

Spoor, Blanche Annie, Matron at Arlington Hospital School Dental Nurse, Northumberland County Council (Royal Infirmary, Newcastle-on-Tyne).

Wynne-Edwards, Helen (Queen Victoria Jubilee Nurses' Institute); Superintendent at Hackney.

---

### DIPLOMAS IN PUBLIC HEALTH.

W. M. Binning, M.R.C.S., L.R.C.P., Univ. Coll.
M. C. Brereton, M.B., Ch.B.Edin., and Univ. Coll.
H. Brookman, M.R.C.S., L.R.C.P., St. George's and Middx.
Alida Charlotte Burt, M.B., B.Ch.Dublin and Univ. Coll.
G. W. Cheater, M.B., B.S.Lond., M.R.C.S., L.R.C.P., Charing Cross and R. Inst. Public Health.
D. Clyde, M.B., Ch.B.Glasgow and Univ. Coll.
E. Cotter, M.B., B.Ch., R.U.I., Dublin, and R.A.M.C.
A. J. Cronin, M.B., Ch.B.Glasgow.
J. A. A. Duncan, L.R.C.P, and S. Edin., L.F.P.S.Glasgow, Edin., and Middx.
G. D. English, M.B., Ch.B.Edin., and R. Inst. Public Health.
Dorothy W. Gowers, M.B., B.S.Lond., M.R.C.S., L.R.C.P., R. Free and Univ. Coll.
C. M. Gwillim, M.B., B.S.Lond., M.R.C.S., L.R.C.P., St. Bart.'s.
G. Habgood, M.R.C.S., L.R.C.P.Camb. and Lond.
C. E. E. Herington, M.B., B.S.Lond., M.R.C.S., L.R.C.P., St. Bart.'s.
A. N. Kingsbury, M.B., B.S.Lond., M.R.C.S., L.R.C.P. Middx.
T. McClurkin, M.B., B.Ch.Belfast, and Univ. Coll.
J. W. Mann, M.B., Ch.B.Aberdeen, and Univ. Coll.
K. Nain, L.M.S.Punjab, and Univ. Coll.
C. C. Okell, M.B., B.Ch.Cantab, M.R.C.S., L.R.C.P. Camb. and Guy's.
R. R. Scott, M.R.C.S., L.R.C.P., Durham and Middx.
J. Singh, M.B., B.S. Punjab and Univ. Coll.
R. M. Soames, M.B., B.C.Cantab, M.R.C.S., L.R.C.P. Camb. and St. Bart.'s.
Elsie Stansfeld, M.B., B.S.Lond., M.R.C.S., L.R.C.P., Cardiff and R. Free.
E. W. L. Thomas, M.B., B.S.Lond., M.R.C.S., L.R.C.P., St. Bart.'s.

---

Now that there are prospects of a real revival of trade, the "Business Efficiency" Exhibition, which will be held at the Central Hall, Westminster, from February 7 to 17, is likely to attract a great deal of attention. The exhibition will be opened at noon on February 7 by Mr. Neville Chamberlain, and all the latest scientific and labour-saving devices will be shown. The exhibits include the latest telephone system, by means of which two persons in different parts of a large building can speak to each other from any part of a room without raising the mouthpiece; the latest calculating machine; and a complete office printer. The exhibition is promoted by the Office Appliance Trades Association.

